# Leprosy cases diagnosed by contacts examination in a hyperendemic capital city of northeastern Brazil^☆^

**DOI:** 10.1016/j.abd.2020.07.016

**Published:** 2021-05-21

**Authors:** Aruse Maria Marques Soares, Rita da Graça Carvalhal Frazão Corrêa, Kezia Cristina Batista dos Santos, Ivan Abreu Figueiredo, Maria de Fátima Lires Paiva, Dorlene Maria Cardoso de Aquino

**Affiliations:** a Department of Nursing, Federal University of Maranhão, São Luís, Maranhão, MA, Brazil; b Department of Collective Health, Universidade Federal do Maranhão, São Luís, Maranhão, MA, Brazil; c Department of Medicine, Universidade Federal do Maranhão, São Luís, Maranhão, MA, Brazil

Dear Editor,

Leprosy, an infectious disease with a prolonged incubation period, has in the household contacts an important means for the maintenance of the endemy. It is characterized by a clinical dermatological-neurological syndrome with a high potential to cause physical disabilities and deformities, in addition to social and psychological impacts.^1^, ^2^

It is still considered a relevant public health problem in most Brazilian states, despite efforts by the Ministry of Health to control the infection.^1^ In 2018, 208,619 new cases were reported worldwide, with Brazil being the 2^nd^ country with the highest prevalence of the disease, registering a total of 28,660 new cases, representing 13.3% of the global total of new occurrences.^3^

Household contacts are highly vulnerable to disease development due to prolonged exposure to the bacilli in the home environment. This risk is approximately five to ten times greater in families with one case of the disease and increases by up to ten times in the event of more than one case in the same household.^2^

In regions of high endemicity, contact surveillance becomes an essential measure for disease control, therefore, it is recommended to carry out a dermatological-neurological evaluation at least once a year, for at least five years, in all contacts. After this period, these contacts should be informed of the possibility of the appearance of signs and symptoms suggestive of leprosy in the future.^4^, ^5^

In 2017, the municipality of São Luís, state of Maranhão, Brazil, was classified as regular in relation to the proportion of examined contacts, as it examined 51.58% of registered contacts, a fact that is considered of concern.^6^ In this sense, knowledge of the occurrence of leprosy cases diagnosed by contact examination among household contacts in that municipality can bring valuable contributions both to the labor practice of health professionals and to users assisted by the Family Health Strategy.

This is an epidemiological, descriptive, retrospective and cross-sectional study with the objective of studying the occurrence of leprosy cases among household contacts of the notified cases in the municipality of São Luís, state of Maranhão, from 2010 to 2017, through information collected from the Notifiable Diseases Information System (SINAN), notification forms and patient records (n = 182).

The data were analyzed using the EPI-INFO program, version 7 (CDC-Atlanta, EUA). The prevalence of leprosy among the examined contacts was calculated, followed by the absolute and relative frequencies for the descriptive analysis. This study is part of the macro-project entitled “Integrated Approach to Clinical, Epidemiological (Space-Temporal), Operational and Psychosocial Aspects of Leprosy in a Hyperendemic Municipality of Maranhão (INTEGRAHANS MARANHÃO)”, approved by the Research Ethics Committee under Register 2.508. 780.

Fig. 1 shows that, during the study period, 17,309 household contacts were registered, 9,387 were examined and 182 were diagnosed with leprosy with a prevalence of 193.9/10,000 contacts. At the beginning of the historical series, 3.52% leprosy cases were notified among contacts, corresponding to the highest percentage. In 2011, there was a decline in the percentage to 2.20%. It was observed that years 2012, 2014, 2015, and 2016 were the periods that showed the lower rates and there were similar percentages in the years 2013 and 2017.

**Figure 1:**
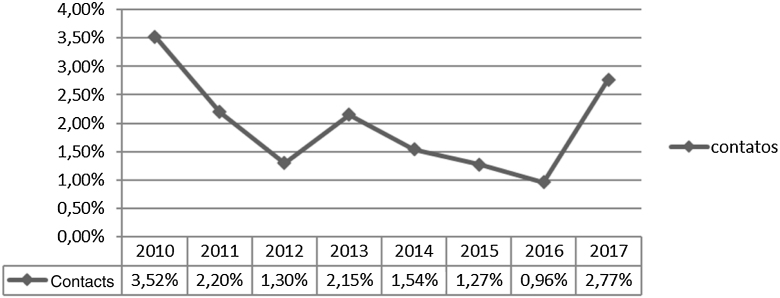
Historical series of leprosy diagnosed by detecting new cases by contact examination per year of notification. São Luís–MA, 2010–2017. Source: SINAN, 2019.

Table 1 shows the sociodemographic characteristics of household contacts diagnosed with leprosy. A higher frequency was observed in the age group of 15–59 years (63.74%), females (56.59%), brown ethnicity (58.79%) and those with incomplete elementary school (26.37%).

**Table 1:** Sociodemographic characteristics of household contacts diagnosed with leprosy. São Luís–MA, 2010–2017.

Variables	n	%
**Age**		
00 to 06 years	11	6.04
07 to 14 years	44	24.18
15 to 59 years	116	63.74
60 to 100 years	11	6.04
**Ethnicity/skin color**		
White	40	22.00
Black	30	16.49
Yellow	01	0.54
Brown	107	58.79
Not recorded	04	2.18
**Sex**		
Male	79	43.41
Female	103	56.59
**Level of schooling**		
Illiterate	07	3.85
1^st^ Grade to incomplete 4^th^ Grade of Elementary School	30	16.50
Complete 4^th^ Grade of Elementary School	09	4.95
5^th^ Grade to incomplete 8^th^ Grade of Elementary School	48	26.37
Complete Elementary School	09	4.95
Incomplete High School	23	12.63
Complete High School	34	18.68
Incomplete Higher Education	05	2.75
Complete Higher Education	03	1.64
Does not apply a/Not recorded	14	7.68
Total	182	100

Source: SINAN, 2019.

a Child aged 0 to 6 years.

Table 2 shows the clinical characteristics: 48.90% of cases corresponded to the borderline form, there was a predominance of a degree of disability equal to zero at the beginning of treatment (64.06%) and bacilloscopy mostly not performed (53.28%). As for the number of lesions and affected nerves, most had up to 5 lesions (87.16%) and no affected nerves (59.34%).

**Table 2:** Clinical characteristics of household contacts diagnosed with leprosy. São Luís–MA, 2010–2017.

Variables	n	%
**Clinical form**		
Indeterminate	27	14.87
Tuberculoid	52	28.57
Bordeline	89	48.90
Lepromatous	10	5.49
Not classified	04	2.20
**Degree of disability**		
Zero	117	64.06
One	47	26.00
Two	08	4.45
Not recorded	10	5.49
**Bacilloscopy**		
Positive	11	6.07
Negative	74	40.65
Not performed	97	53.28
**Number of lesions**		
Up to 5 lesions	155	87.16
06 to 10 lesions	15	8.26
+ than 10 lesions	07	3.84
Not recorded	05	2.74
**Number of affected nerves**		
None	108	59.34
01 to 04	53	29.14
more than 04	19	10.43
Not recorded	02	1.09
Total	182	100

Source: SINAN, 2019.

Fig. 2 shows that the borderline clinical form was predominant in all years, with the exception of 2012, in which 50.00% of the cases corresponded to the tuberculoid form. Variations were also observed in the percentages of the indeterminate, tuberculoid and lepromatous forms during the years 2010 to 2017.

**Figure 2:**
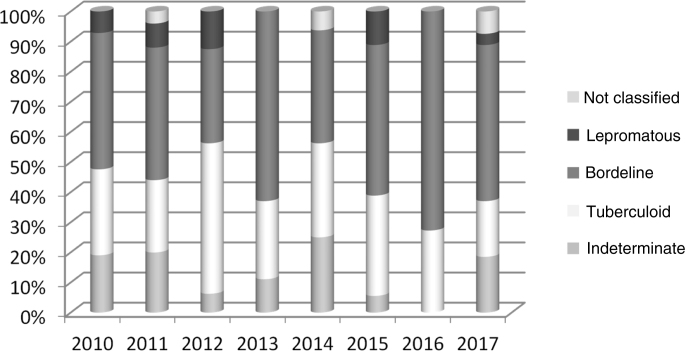
Clinical form in the household contacts diagnosed with leprosy per year of notification. São Luís–MA, 2010–2017. Source: SINAN, 2019.

The prevalence of contacts with leprosy was higher than the results of a similar survey also carried out in São Luís, state of Maranhão.^2^ The high percentage of prevalence found in the municipality is directly related to the low coverage of the Family Health Strategy (FHS) in São Luís, which is only 34.67%. Municipalities with low coverage can limit the actions of the FHS teams, especially regarding surveillance and contact control actions.^7^

The predominant age group (15–59 years) is a matter of concern, as it includes young individuals of productive age, with a negative impact on the labor market and the national, family and individual economy.^8^ It is also noteworthy that 30.2% of new leprosy cases diagnosed among the examined contacts were children and adolescents under the age of 15, which is a major epidemiological concern in relation to disease control in the municipality.

The low level of schooling found among the individuals may be related to the lack of information about the disease and its particularities and, consequently, the delay in seeking adequate assistance in health services, making the diagnosis and adherence to treatment difficult.^9^

The borderline clinical form indicates late diagnosis and failure in Primary Care actions developed for this purpose. Other national studies have found similar results, in places where the multibacillary forms prevailed over paucibacillary ones.^1^, ^2^, ^4^, ^10^

There was a predominance of zero degree physical disability; however, there were cases with grades 1 and 2. It was observed there is a contradiction between the predominance of the borderline clinical form and the zero degree physical disability. This fact can be attributed to the lack of preparation and/or experience to carry out the assessment of the degree of disability or some technical or operational problems when sending data to SINAN.

The study has limitations regarding the use of secondary data, due to possible errors or absence of information in the medical records, notification forms and SINAN system. The sociodemographic and clinical characteristics found in this study reflect the fact that leprosy is a neglected disease and the precariousness of actions aimed at early detection among contacts, demonstrated by the high prevalence rate of leprosy among household contacts and the predominant borderline clinical form.

## Financial support

This study received financial support from the 10.13039/501100003758Foundation for the Support of Research and Scientific and Technological Development of Maranhão(FAPEMA, *Fundação de Amparo à Pesquisa e ao Desenvolvimento Científico e Tecnológico do Maranhão*, process n. 01347/17).

The funding source had no involvement in the study design selection process; in the collection, analysis and interpretation of data; writing of the report; and in the decision to send the article for publication.

## Authors’ contributions

Aruse Maria Marques Soares: Approval of the final version of the manuscript; design and planning of the study; drafting and editing of the manuscript; collection, analysis, and interpretation of data; critical review of the literature; critical review of the manuscript.

Rita da Graça Carvalhal Frazão Corrêa: Approval of the final version of the manuscript; design and planning of the study; critical review of the manuscript.

Kezia Cristina Batista dos Santos: Approval of the final version of the manuscript; drafting and editing of the manuscript; critical review of the manuscript.

Ivan Abreu Figueiredo: Approval of the final version of the manuscript; critical review of the manuscript.

Maria de Fátima Lires Paiva: Approval of the final version of the manuscript; critical review of the manuscript.

Dorlene Maria Cardoso de Aquino: Approval of the final version of the manuscript; design and planning of the study; drafting and editing of the manuscript; collection, analysis, and interpretation of data; effective participation in research orientation; critical review of the manuscript.

## Conflicts of interest

None declared.
